# Maximum Constrained Directivity of Oversteered End-Fire Sensor Arrays

**DOI:** 10.3390/s150613477

**Published:** 2015-06-09

**Authors:** Andrea Trucco, Federico Traverso, Marco Crocco

**Affiliations:** 1 Department of Electrical, Electronic, Telecommunications Engineering, and Naval Architecture (DITEN), University of Genoa, 5-16126 Genova, Italy; E-Mail: federico.traverso@ginevra.dibe.unige.it; 2 Pattern Analysis & Computer Vision (PAVIS), Istituto Italiano di Tecnologia (IIT), 30.16163 Genova, Italy; E-Mail: marco.crocco@iit.it

**Keywords:** beamforming, end-fire arrays, oversteering, maximum directivity, white noise gain, microphone and hydrophone arrays

## Abstract

For linear arrays with fixed steering and an inter-element spacing smaller than one half of the wavelength, end-fire steering of a data-independent beamformer offers better directivity than broadside steering. The introduction of a lower bound on the white noise gain ensures the necessary robustness against random array errors and sensor mismatches. However, the optimum broadside performance can be obtained using a simple processing architecture, whereas the optimum end-fire performance requires a more complicated system (because complex weight coefficients are needed). In this paper, we reconsider the oversteering technique as a possible way to simplify the processing architecture of equally spaced end-fire arrays. We propose a method for computing the amount of oversteering and the related real-valued weight vector that allows the constrained directivity to be maximized for a given inter-element spacing. Moreover, we verify that the maximized oversteering performance is very close to the optimum end-fire performance. We conclude that optimized oversteering is a viable method for designing end-fire arrays that have better constrained directivity than broadside arrays but with a similar implementation complexity. A numerical simulation is used to perform a statistical analysis, which confirms that the maximized oversteering performance is robust against sensor mismatches.

## 1. Introduction

Data-independent beamforming is a well-known array signal processing method characterized by a moderate computational burden [1,2], which is frequently applied in low-cost, small-sized linear arrays with a fixed steering direction. These arrays are useful in numerous electronic systems for spatially processing far-field acoustic waves, such as microphone arrays for sound capture and voice-based interactions [3,4,5,6] and hydrophone arrays for underwater acoustic monitoring and communications [7,8].

**Figure 1:**
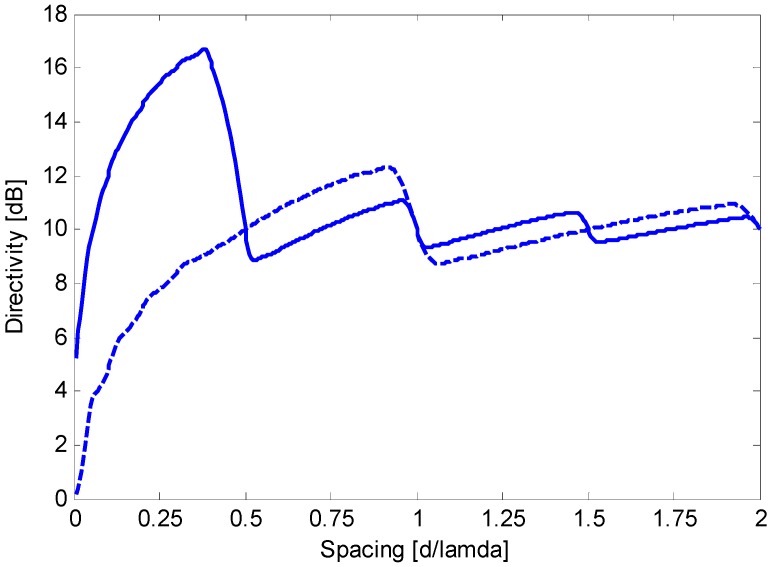
Maximum constrained directivity obtained for a 10-element array *versus* the normalized inter-element spacing (*i.e.*, *d*/λ) for end-fire (solid line) and broadside (dashed line) steering. Optimum weights are computed by imposing WNG ≥ 0 dB and solving Equation (23).

**Figure 2:**
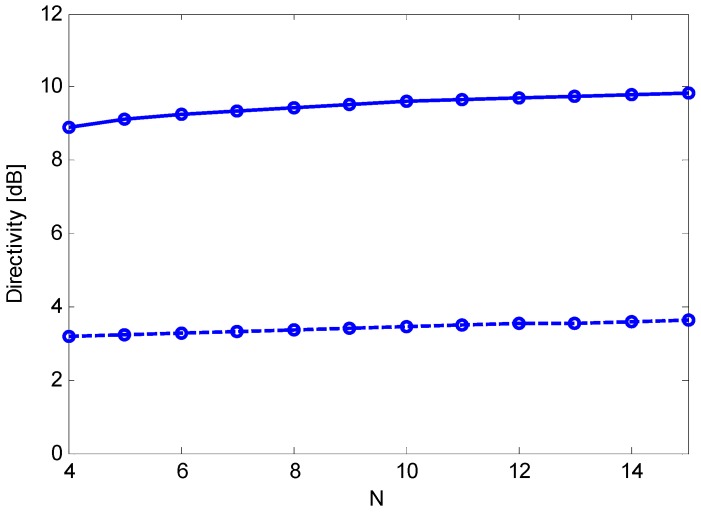
Maximum constrained directivity obtained for an array aperture of *D* = 0.45 λ *versus* the number of sensors, *N*, for end-fire (solid line) and broadside (dashed line) steering; Optimum weights are computed by imposing WNG ≥ 0 dB and solving Equation (23).

In this context, end-fire steering is frequently preferred to broadside steering [3,4,6] because it enables a significantly higher directivity. In principle, an end-fire array can attain a directivity of *N*^2^, where *N* is the number of sensors [9]. However, to limit the sensitivity to random array errors and sensor mismatches, a constraint on the sensitivity factor, *i.e.*, the inverse of the white noise gain (WNG), can be introduced [2,10,11]. Because random errors and mismatches are uncorrelated between sensors, they serve as spatially white noise [2,10,11]. As a result, the constrained directivity maximization is a classical approach to achieve robust superdirective performance via end-fire arrays [2,10,11]. Although the WNG constraint prevents a directivity of *N*^2^, the maximum constrained directivity is often significantly greater than *N* and is attained for a value of the inter-element spacing *d* that is greater than zero and smaller than λ/2, where λ is the wavelength. Figure 1 illustrates the maximum constrained directivity *versus* the inter-element spacing for end-fire and broadside arrays, when *N* = 10 and WNG ≥ 0 dB. Figure 2 illustrates the maximum constrained directivity *versus* *N* for the two steering directions when *D* = 0.45 λ, where *D* is the array aperture and when WNG ≥ 0 dB.

Despite its advantages, end-fire steering requires a more complex processing structure than broadside steering. To achieve the maximum constrained directivity of an end-fire array, complex-valued weight coefficients (in this paper the weight coefficients do not include the phase terms that correspond to the time delays required to steer the main lobe at end-fire) should be applied. A typical delay-and-sum beamforming structure [1,2] must be doubled to process both the real part and the imaginary part of the signals. In addition, the real signals generated by the array sensors must be converted to their complex analytic versions prior to processing. To simplify the processing system, an alternative is the processing of real signals, which are scaled using the moduli of the weight coefficients and opportunely delayed. The delays that are required to steer the main lobe at end-fire must be updated based on the phases of the weight coefficients. This alternative of the complex processing is only valid for narrow-band signals. Moreover, the updated delays are not integer multiples of a given time interval; consequently, their implementation in digital electronic systems is challenging.

**Figure 3:**
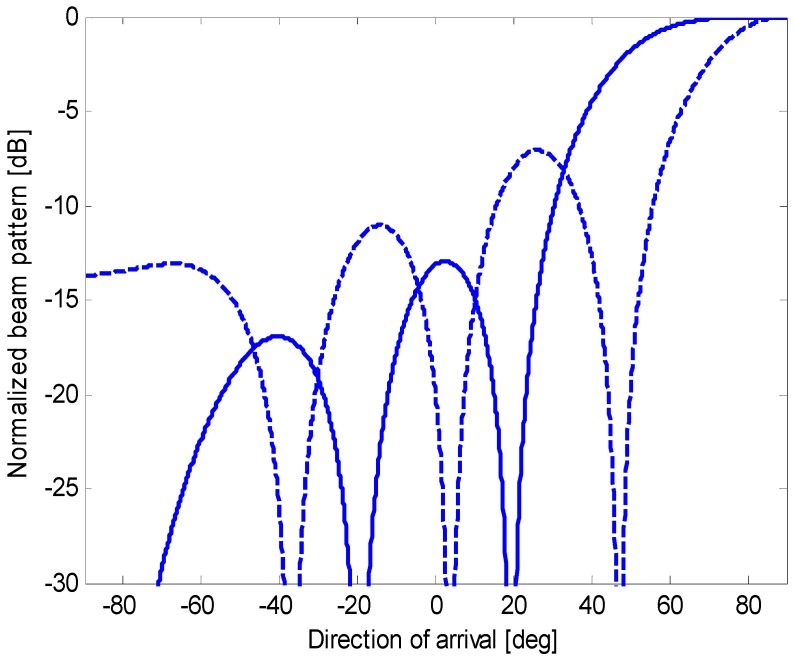
Normalized end-fire beam patterns for an array of 10 sensors with a spacing *d* = 0.15 λ without oversteering (solid line) and with oversteering (dashed line), using a uniform weighting window.

In this paper, we demonstrate that an oversteered end-fire array, if adequately optimized, yields a constrained directivity that is similar to the maximum directivity (achieved by complex weight coefficients) and circumvents the previously mentioned implementation difficulties. Oversteering [3,4,5,7,10,12,13] is a technique applied to increase the directivity of an end-fire array by pushing its main-lobe peak past the end-fire, outside the visible region. The main lobe is steered *past the end-fire* (or *oversteered*) by inserting additional delays. If the sensors are spaced less than λ/2 and the beam pattern shift is adequately tuned, a reduction in the width of the main lobe in the visible region is obtained and the appearance of grating lobes is avoided. Unfortunately, the main-lobe absolute level is reduced, whereas the absolute levels of the side lobes are not modified. Thus, as shown in Figure 3, the oversteering operation reduces the main-lobe width but increases the side-lobe level relative to the main-lobe [10]. In particular, if the weight coefficients are held constant, a gradual increase in the oversteering amount causes a progressive decrease in the WNG (this statement can be easily demonstrated by the equation that defines the WNG, which is introduced in Section 2) (*i.e.*, a robustness reduction).

For these reasons, the oversteering amount is typically carefully set [12] or tuned [5,7,10] to avoid exceeding the “mild oversteering” extent. In addition, real-valued weight coefficients that are traditionally used to reduce side lobes (especially the Taylor window) are also adopted in this context [5,7,10].

The contribution of this paper is twofold. First, we propose a mathematical framework that enables the computation of the complex weight coefficients and real weight coefficients that maximize the constrained directivity for end-fire arrays. The merit of this framework, which we preliminary presented in [14], is the ability to consider oversteering. This framework enables the performance of delay-and-sum end-fire arrays (with optimum complex or real weight coefficients) to be compared with the performance of oversteered arrays (with traditional weighting windows or optimum real-valued weight coefficients).

Second, we propose an optimization algorithm that determines the oversteering amount and the real-valued weight coefficients to maximize the constrained directivity for a given inter-element spacing and a given lower bound for the WNG value. For the computation of the weight coefficients, the proposed algorithm exploits the previously mentioned framework.

The contribution of this paper is innovative because the oversteering literature does not attempt to optimize the oversteering amount and does not propose any method to compute the weight coefficients that maximize the directivity by satisfying a lower bound for the WNG value. The synthesis of a weighting window that considers the presence of a given oversteering amount has only been addressed in [13]. However, only a sub-optimal, specific technique, devoted to reduce the side-lobe level, is proposed in that study. In addition, this paper demonstrates that when oversteering is adopted for an equally spaced (ES) linear array and a suitable sampling frequency is established, all delays to be applied to the received signals are integer multiples of the sampling period. This fact dramatically simplifies the system architecture. Because the WNG constraint assures robust performance against sensor mismatches [2,10,11], the opportunity for using low-cost sensors is provided. This robustness is statistically assessed by assuming a microphone array and simulating numerous realizations for the sensor characteristics.

This paper is organized as follows: Section 2 provides background information on beamforming. Section 3 presents the framework to compute the weights that maximize the constrained directivity and the algorithm for the oversteering optimization. In Section 4, the obtained directivities *versus* the spacing are discussed, some implementation issues are explored, and the robustness is assessed using numerical simulations. Finally, the conclusions are presented in Section 5.

## 2. Beamforming and Oversteering

Let us consider an ES linear array composed of *N* omnidirectional point-like sensors that is centered at the coordinate origin and placed on the *x* axis (because fixed steering is considered, if we assume that the array is composed of finite-sized transducers pointed at the array end-fire, the main lobe magnitude is not altered and the side lobe magnitude is only marginally reduced. However, the directivity increase is negligible because the small inter-element spacing considered here strictly limits the transducer aperture). The *n*th sensor is placed at the position *x_n_* and generates the signal *s_n_*(*t*), which is proportional to the sum of the desired and noise wavefields. According to conventional delay-and-sum beamforming [1,2], the beam signal *b*(*t*, θ_0_) steered in the direction θ_0_ is computed as:

(1) $$b\left(t,\theta_{0}\right)=\sum_{n=1}^{N}\;w_{n}\;s_{n}\left(t-\tau_{n}\right)$$

where *t* is the time and *w_n_* is the weight coefficient associated with the *n*th sensor. For far-field plane waves, the delay τ*_n_* is computed as:

(2) $$\tau_{n}=x_{n}\sin\theta_{0}/c=x_{n}u_{0}/c$$

where *c* is the wave propagation speed and the angle θ_0_ is measured with respect to the *y* axis (*i.e.*, θ_0_ = 90° at end-fire), and *u*_0_ = sinθ_0_.

If the weight coefficients *w_n_* are real values, the real signals generated by the sensors can be directly employed as the input signals *s_n_*(*t*). If the weight coefficients *w_n_* are complex values, the input signals *s_n_*(*t*) are complex analytic signals computed from the real signals generated by the sensors; the real part of the analytic beam signals obtained from the beamforming output can then be employed as the final result. However, if the input signals have a narrow-band spectrum centered at the frequency *f*, an equivalent result is obtained by working with the real signals and using the phases φ*_n_* of the weight coefficients *w_n_* as additional delays for the signals *s_n_*(*t*) as follows:

(3) $$b\left(t,\theta_{0}\right)=\sum_{n=1}^{N}\;\left|w_{n}\right|\;s_{n}\left(t-\tau_{n}+\frac{\phi_{n}}{2\pi f}\right)$$

Unlike conventional delay-and-sum beamforming, when the oversteering technique is applied, an additional delay $\tau_{n}^{o}$ is introduced to steer the main lobe past end-fire. The beamforming equation becomes:

(4) $$b\left(t,\theta_{0}\right)=\sum_{n=1}^{N}w_{n}s_{n}\left(t-\tau_{n}+\tau_{n}^{o}\right)$$

(5) $$\tau_{n}^{o}=x_{n}\varepsilon /c$$

where ε is the amount of oversteering, which rules the additional delay and is expressed in a scale that is comparable with *u*_0_ = sinθ_0_. A better physical understanding of ε will be possible after the introduction of the beam pattern function. At this stage, we observe that the weight coefficients are real values and the input signals and output beam signal are real functions in both Equations (3) and (4). The total delays to be applied to the *n*th signal are determined for the two cases as:

(6) $$\tau_{n,cmp}=\tau_{n}-\frac{\varphi_{n}}{2\pi f}=\frac{x_{n}-\varphi_{n}/k}{c}$$

(7) $$\tau_{n,ovs}=\tau_{n}+\tau_{n}^{o}=\frac{x_{n}\left(1+\varepsilon \right)}{c}$$

where τ*_n,cmp_* is the total delay to be used in Equation (3), τ*_n,ovs_* is the total delay to be used in Equation (4), and *k* is the wavenumber, *k* = 2π*f*/*c*.

For a plane wave of frequency *f* and direction of arrival θ, the resulting complex beam pattern *B*(*u*) is expressed as:

(8) $$B\left(u\right)=\sum_{n=1}^{N}\;w_{n}\text{ exp}\left[jkx_{n}\left(u-u_{0}-\varepsilon \right)\right]$$

where *u* = sinθ. This expression is valid for both real-valued and complex-valued weight coefficients *w_n_*. Moreover, it can be used for end-fire steering (*u*_0_ = 1) with or without oversteering (depending on the value of ε), as well as for any other steering (*u*_0_ ≠ 1 and ε = 0).

In conventional end-fire steering, the main-lobe peak occurs at *u* = *u*_0_ = 1. When an amount of oversteering is introduced, the main-lobe peak occurs at *u* = *u*_0_ + ε = 1 + ε, *i.e.*, which is outside the visible region *u* ϵ [–1,1]. Consequently, the main-lobe portion that remains inside the visible region is narrower than the main-lobe in conventional end-fire, as shown in Figure 3. This result is obtained using the additional delays that are defined in Equation (5). They act similarly to the phases φ*_n_* of the complex weights in Equation (3) but with fewer degrees of freedom (*i.e.*, only one variable –ε– instead of *N* variables –φ*_n_*). In the frequency domain, the additional delays used to oversteer the main lobe are equivalent to the addition of the phase terms that are linear with the sensor position. In some cases [10], the linear-phase adequately approximates the phase of the complex weights, and similar performances are obtained.

The directivity *D* of a linear array steered in the direction θ_0_ is defined [2] as follows:

(9) $$D=\frac{{\left|B\left(u_{0}\right)\right|}^{2}}{\frac{1}{2}\int_{-1}^{1}{\left|B\left(u\right)\right|}^{2}du}$$

By substituting Equation (8) in Equation (9), the following equation is obtained after some mathematics:

(10) $$D=\frac{\sum_{m=1}^{N}\;\sum_{n=1}^{N}\;\bar{w_{m}}w_{n}\text{ exp}\left[jk\left(x_{m}-x_{n}\right)\varepsilon \right]}{\sum_{m=1}^{N}\;\sum_{n=1}^{N}\;\bar{w_{m}}w_{n}\text{ exp}\left[jk\left(x_{m}-x_{n}\right)\left(u_{0}+\varepsilon \right)\right]\text{ sinc}\left[k\left(x_{n}-x_{m}\right)/\pi \right]}$$

where sinc(η) = sin(πη)/(πη) and $\bar{w_{m}}$ is the complex conjugate of *w_m_*. For the same array, the WNG *G_W_* is defined [2] and computed as follows:

(11) $$G_{W}=\frac{{\left|B\left(u_{0}\right)\right|}^{2}}{\sum_{n=1}^{N}{\left|w_{n}\right|}^{2}}=\frac{\sum_{m=1}^{N}\;\sum_{n=1}^{N}\;\bar{w_{m}}w_{n}\text{ exp}\left[jk\left(x_{m}-x_{n}\right)\varepsilon \right]}{\sum_{n=1}^{N}{\left|w_{n}\right|}^{2}}$$

The directivity and WNG can be rewritten in matrix form by defining a column vector **w** for which the *n*th element is **w**[*n*] = *w_n_*, *n* = 1, 2, …, *N*, a square matrix **A** of size *N* for which the element in the *m*th row and *n*th column is:

(12) $$\text{A}\left[m,n\right]=\exp[jk(x_{m}-x_{n})(u_{0}+\varepsilon )]\mathrm{sinc}[k(x_{n}-x_{m})/\pi ]$$

and a square matrix **B** of size *N* for which the element in the *m*th row and *n*th column is:

(13) $$\text{B}\left[m,n\right]=\exp[jk(x_{m}-x_{n})\varepsilon ]$$

With this notation, the directivity and WNG are defined as:

(14) $$D=\frac{w^{*}\;B\;w}{w^{*}\text{ A }w}$$

(15) $$G_{W}=\frac{w^{*}\;B\;w}{w^{*}\;w}$$

where * indicates the complex-conjugate transpose.

### Properties of Matrix **A**

Regarding matrix **A**, because w*Aw is equal to the integral of the beam pattern modulus squared, w*Aw is greater than zero for all non-zero complex vectors **w**. Consequently, **A** is a positive-definite matrix, implying that **A** is a Hermitian matrix. Therefore, the real part of **A** is a symmetric and positive-definite real matrix, whereas the imaginary part is a skew-symmetric real matrix (*i.e.*, **w***^T^*Im{**A}w** = 0 for all real vectors **w**, where *^T^* indicates the transpose).

## 3. Optimization Method

First, we consider the well-known maximum directivity problem without any constraint on the WNG. Our goals are to encompass the presence of oversteering and to emphasize the symmetry properties of the weight vector toward proposing a method to compute the real weight coefficients that provide the maximum constrained directivity. The proofs of these symmetry properties are essential for demonstrating that the related weight vectors maximize the directivity. In addition, the unconstrained optimization is a valuable background for approaching the constrained optimization.

### 3.1. Directivity Maximization by Weight Coefficients

Assuming, without loss of generality, a unitary response in the steering direction:

(16) $$\text{B}(u_{0})=\sum_{n=1}^{N}\;w_{n}\text{exp(}-jkx_{n}\varepsilon \text{)}=1$$

the numerator in Equations (14) and (15) becomes one because w*B w = |*B*(*u*_0_)|^2^. Therefore, the problem of finding vector **w** that maximizes the directivity can be expressed in the form:

(17) $$\begin{array}{l} \underset{w}{\text{Minimize}}\;w^{*}\;A\;w\\ \text{subject to }b^{*}\;w\;=\text{ 1 } \end{array}$$

where **b** is a column vector for which the *n*th element is **b**[*n*] = exp(*jkx_n_*ε) and b*w = *B*(*u*_0_). By using the method of Lagrange multipliers (because **A** is positive definite and the Lagrangian function is real-valued [15]), it can be concluded that for a given oversteering amount ε, the solution **w***_o_* is expressed as follows:

(18) $$w_{o}=\frac{A^{-1}\;b}{b^{*}\;A^{-1}\;b}$$

**Proposition 1.** *For an ES linear array, the complex weight coefficients **w**_o_ that solve Equation (17) are conjugate symmetric with respect to the array center*.

**Proof.** When the discrete antenna is an ES linear array, matrix **A** is a Toeplitz matrix. Because **A** is positive definite, its inverse, **A**^−1^, is also positive definite. Moreover, because **A** is a Hermitian and Toeplitz matrix, its inverse is a Hermitian and persymmetric matrix [16]. As an example, let us denote the element (*m*, *n*) of matrix **A**^−1^ as *a_mn_* (*i.e.*, *a_mn_* = **A**^−1^[*m*, *n*]) and set *N* = 5. Due to the aforementioned symmetry properties, matrix **A**^−1^ has the following form:

(19) $$A^{-1}=\left[\begin{array}{ccccc} a_{11} & a_{12} & a_{13} & a_{14} & a_{15}\\ \bar{a_{12}} & a_{22} & a_{23} & a_{24} & a_{14}\\ \bar{a_{13}} & \bar{a_{23}} & a_{33} & a_{23} & a_{13}\\ \bar{a_{14}} & \bar{a_{24}} & \bar{a_{23}} & a_{22} & a_{12}\\ \bar{a_{15}} & \bar{a_{14}} & \bar{a_{13}} & \bar{a_{12}} & a_{11} \end{array}\right]$$

Now, let us denote the *n*th element of vector **b** as *b_n_, i.e.*, *b_n_* = **b**[*n*]. Assuming that the ES linear array is centered at the coordinate origin, vector **b** has the following form:

(20) $$b=\left[\begin{array}{c} b_{1}\\ b_{2}\\ 0\\ \bar{b_{2}}\\ \bar{b_{1}} \end{array}\right]$$

Because all of the elements on the main diagonal of any Hermitian matrix are necessarily real, it is easy to verify that the product **A**^−1^**b** produces a vector that is conjugate symmetric with respect to its center. Finally, because we know that b*A^−1^**b** is a real number greater than zero, the solution **w***_o_* in Equation (18) is a complex, conjugate-symmetric vector. Although this fact has been demonstrated for odd values of *N*, it is straightforward to verify that the same conclusion holds for even values of *N*.

In the special case of broadside steering (*i.e.*, *u*_0_ = 0 and ε = 0), matrix **A** and vector **b** are both real, so the optimum weights in vector **w***_o_* are real valued and symmetric. If complex weights must be avoided, the vector of real-valued weight coefficients that solves the problem in Equation (17) should be computed.

**Proposition 2.** *For an ES linear array, the real weight coefficients **w**_oR_ that solve Equation (17) are symmetric with respect to the array center, and vector **w**_oR_ is given by:*

(21) $$w_{oR}=\frac{A_{R}^{-1}\;b_{R}}{b_{R}^{T}\;A_{R}^{-1}\;b_{R}}$$

*where **A**_R_ = Re{**A}** and **b**_R_ = Re{**b}***.

**Proof.** Because we know that **w***^T^* Im{**A}w** = 0 for all real vectors **w**, to solve Equation (17) it is sufficient to minimize **w***^T^***A***_R_***w**. Regarding the constraint, to assure b*w = 1 with a real vector **w**, it is necessary that Re{b*}w = 1 and Im{b*}w = 0. Therefore, the optimization problem in Equation (17) can be rewritten as follows:

(22) $$\begin{array}{l} \underset{w}{\text{Minimize}}\;w^{T}\;A_{R}\;w\\ \text{subject to }{b_{R}}^{T}\;w\;=\text{ 1 } \end{array}$$

if the solution of this problem, **w***_oR_*, verifies the equation Im{b*}w*_oR_* = 0. Because **A***_R_* is positive definite and the Lagrangian function is real, for a given oversteering amount ε, the solution **w***_oR_* for Equation (22) is provided in Equation (21). Moreover, for any ES linear array, matrix **A***_R_* is a symmetric and Toeplitz matrix, with an inverse that is a symmetric and persymmetric matrix [16]. Analogous to the proof of Proposition 1, it is possible to verify that vector **w***_oR_* is symmetric with respect to its center. Therefore, due to the conjugate symmetry of **b** and the symmetry of **w***_oR_*, the equation Im{b*}w*_oR_* = 0 is verified for any solution **w***_oR_*.

### 3.2. Constrained Directivity Maximization by Weight Coefficients

The design of robust solutions for maximum-directivity arrays requires the introduction of a constraint on the WNG. The WNG constraint is not only used for data-independent beamforming [2,10,11]; it has also been used to improve the robustness of data-dependent beamforming techniques, e.g., the norm-constrained Capon beamforming [17,18,19]. The introduction of this constraint prohibits the use of the analytical solutions in Equations (18) and (21). The problem in Equation (17) should be rewritten as follows:

(23) $$\begin{array}{l} \underset{w}{\text{Minimize}}\;w*\;A\;w\\ \text{subject to }\left\{ \begin{array}{l} w*\;w\;\leq \;G_{th}^{-1}\\ b*\;w\;=\text{ 1} \end{array}\right.\; \end{array}$$

Here, *G_th_* is the lower bound for the WNG value, *i.e.*, *G_W_* ≥ *G_th_*. Because the WNG value cannot exceed the number of sensors (*G_W_* ≤ *N*), the lower-bound *G_th_* should be appropriately established, *i.e.*, 0 < *G_th_* ≤ *N*. The weights *w_n_* = (1/*N*)exp(*jkx_n_*ε) satisfy b*w = 1 and yield the maximum WNG, *i.e.*, *G_W_* = *N*. Therefore, for every ε and every *G_th_*, 0 < *G_th_* ≤ *N*, at least one complex vector exists that solves Equation (23).

This optimization problem is a quadratically constrained quadratic program that can be solved by a convex optimization tool (e.g., CVX, a package for specifying and solving convex programs [20]). In general, the solution is a vector **w***_o_* of complex weight coefficients. The introduction of a convex inequality constraint on the norm squared of vector **w***_o_* does not alter the solution symmetry properties discussed in Proposition 1.

*Proposition 3*: For an ES linear array, the complex weight coefficients **w***_o_* that solve Equation (23) are conjugate symmetric with respect to the array center.

*Proof*: To demonstrate this proposition, let us introduce the Lagrangian [15] for the problem in Equation (23):

(24) $$\;L\left(w,\lambda ,\alpha \right)=w^{*}\;A\;w+\lambda \left(w^{*}w-G_{th}^{-1}\right)+\alpha \left(b^{*}\;w-1\right)$$

where α and λ are real-valued Lagrange multipliers, with α being arbitrary and λ ≥ 0 due to the inequality constraint. By forcing the gradient of the Lagrangian function to be equal to zero:

(25) $$\nabla_{w}\;L\left(w,\lambda ,\alpha \right)=0$$

where **0** is a column vector of size *N*, the solution for the problem in Equation (23) is obtained as a function of α and λ:

(26) $$w_{o}=-\frac{\alpha }{2}{\left(A+\lambda I\right)}^{-1}\;b$$

where **I** is an *N* × *N* identity matrix. By imposing the equality constraint b*w*_o_* = 1, the solution in Equation (26) becomes:

(27) $$w_{o}=\frac{{\left(A+\lambda I\right)}^{-1}\;b}{{\text{b}}^{\text{*}}{\left(A+\lambda I\right)}^{-1}\;b}$$

Because λ ≥ 0, A + λI and its inverse are definite positive. Moreover, because **A** is a Hermitian and Toeplitz matrix, A+λI is also a Hermitian and Toeplitz matrix. Therefore, ${\left(A\;+\;\lambda I\right)}^{-1}$ is a Hermitian and persymmetric matrix [16]. Following the same reasoning as in the proof of Proposition 1, **w***_o_* is again a complex, conjugate-symmetric vector.

The similarity between Equation (27) and the optimum weight vector equation in norm-constrained Capon beamforming [17,18,19] is evident. In the latter technique, the matrix **A** is replaced by the covariance matrix of the array signals and the oversteering is not included. The data-dependent solutions are equivalent if the oversteering amount is zero and an isotropic noise field is assumed to be the only signal that impinges on the array.

The general formulation in Equation (23) can be adapted to the specific cases addressed in this paper. The computation of the optimum complex-valued weights is of interest when oversteering is not active (*i.e.*, ε = 0 and all of the elements of vector **b** are equal to one). In contrast, the computation of a solution composed of real weights may be of interest either with or without oversteering.

However, for a given ε, ε > 0, the maximum WNG value that is achievable by real weights is less than *N*. The maximum *G_R_*_max_ is dependent on ε and is equivalent to *G_R_*_max_(ε) = **b***_R_^T^* **b***_R_*. Therefore, a real solution for Equation (23) only exists if *G_th_* ≤ *G_R_*_max_(ε).

*Proposition 4*: For an ES linear array, if the real vector **w***_oR_* that solves Equation (23) exists, the weight coefficients in **w***_oR_* are symmetric with respect to the array center and vector **w***_oR_* can be computed by solving the following problem:

(28) $$\begin{array}{l} \underset{w}{\text{Minimize}}\;w^{T}\;A_{R}\;w\\ \text{subject to }\left\{ \begin{array}{l} w^{T}\;w\;\leq \;G_{th}^{-1}\\ {b_{R}}^{T}\;w\;=\text{ 1} \end{array}\right.\; \end{array}$$

*Proof*: Because we know that **w***^T^* Im{**A}w** = 0 for all real vectors **w**, to solve Equation (23) it is sufficient to minimize **w***^T^***A***_R_***w**. Regarding the equality constraint, to assure b*w = 1 with a real vector **w**, it is necessary that Re{b*}w = 1 and Im{b*}w = 0. Therefore, the optimization problem in Equation (23) can be rewritten as in Equation (28) if the solution **w***_oR_* of this new problem satisfies the equation Im{b*}w*_oR_* = 0. By applying the identical reasoning as the proof of Proposition 3, it can be verified that the solution of Equation (28) is:

(29) $$w_{oR}=\frac{{\left(A_{R}+\lambda I\right)}^{-1}\;b_{R}}{\;{b_{R}}^{\text{T}}{\left(A_{R}+\lambda I\right)}^{-1}\;b_{R}}$$

For any ES linear array, matrix $A_{R}\;+\;\lambda I$ is a symmetric and Toeplitz matrix, with an inverse that is a symmetric and persymmetric matrix [16]. Analogous to the proof of Proposition 1, it is possible to verify that vector **w***_oR_* is symmetric with respect to its center. Therefore, due to the conjugate symmetry of **b** and the symmetry of **w***_oR_*, the equation Im{b*}w*_oR_* = 0 is verified for any solution **w***_oR_*. Similar to Equations (23) and (28) this can be solved using a convex optimization tool [20], which obtains a real vector **w***_oR_*.

### 3.3. Oversteering Optimization

When the oversteering is applied and real weight coefficients are desired, the constrained directivity must be maximized with respect to both **w** and ε because the elements of **A***_R_* and **b***_R_* are dependent on ε. Unlike the optimization with respect to **w**, the optimization with respect to ε does not have an analytical solution. A feasible approach is to compute the optimum vector **w***_oR_* for each possible value of ε, which is referred to as **w***_oR_*(ε), and to select the couple [ε, **w***_oR_*(ε)] that provides the highest constrained directivity. To make this exhaustive search possible, the allowable domain of ε, which depends on the ratio λ/*d*, should be adequately discretized.

In principle, the upper bound for the oversteering amount is dependent on the sensor spacing: it corresponds to the beam-pattern shift that brings the grating lobe from the outside of the visible region to the inside. To prevent a grating lobe at θ= −90°, the allowable domain for the oversteering amount ε is the interval (0, λ/*d* − 2). If the inter-element spacing *d* is decreased, the extent of the domain of ε increases.

Note that the problem in Equation (23) can be solved by a real weight vector only if *G_th_* ≤ *G_R_*_max_(ε), where ε ϵ (0, λ/*d* − 2).

Based on these considerations, the total optimization algorithm for **w** and ε can be summarized as follows:

1. A discretization step is defined for ε and the interval (0, λ/*d* − 2) is discretized accordingly (a beam-pattern shift due to a given ε has an impact on the directivity value that depends on the main-lobe width. Because we desire that a change of ε equal to its discretization step will produce a small directivity change, such a step must be much smaller than the main-lobe width). The discrete values of ε are indicated by ε*_k_*.
2. The values ε*_k_* for which the inequality *G_th_* ≤ *G_R_*_max_(ε*_k_*) is satisfied are determined and indicated by ε*_z_*.
3. For each ε*_z_*, the optimum weight vector **w***_oR_*(ε*_z_*) that maximizes the constrained directivity is computed by solving Equation (28).
4. For each couple [ε*_z_*, **w***_oR_*(ε*_z_*)], the related directivity is computed by Equation (14).
5. The couple [ε*_z_*, **w***_oR_*(ε*_z_*)] that provides the highest directivity is selected, and the related directivity value represents the maximum for the constrained directivity.

## 4. Results and Discussion

### 4.1. Directivity Versus Spacing

Initially, a linear array composed of eight ES sensors is considered, and the WNG is imposed to be always ≥ 0 dB. The directivity of this array is evaluated as a function of the normalized spacing (*i.e.*, the ratio between *d* and λ, *d*/λ) considering spacing values smaller than λ/2. Regarding oversteering, three different real weighting windows are considered: uniform, Taylor’s, and the optimum window computed by solving Equation (28).

**Figure 4:**
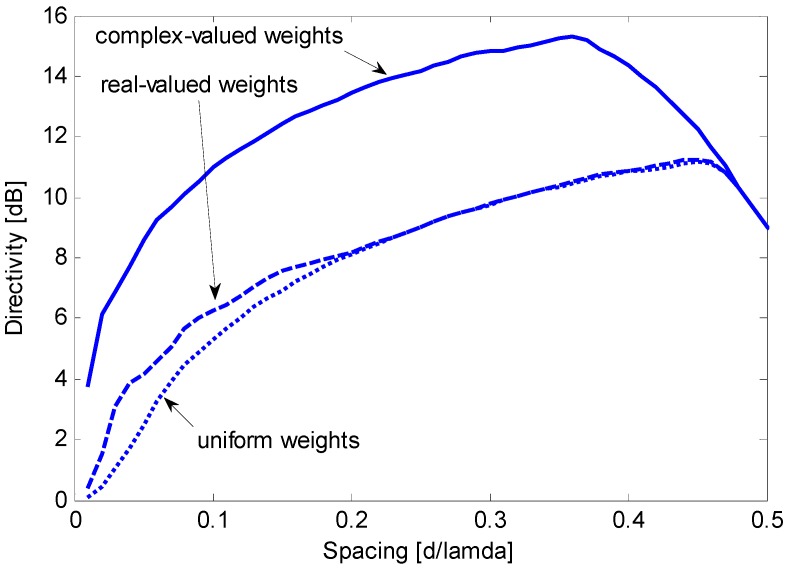
Maximum constrained directivity obtained for an end-fire array of *N* = 8 sensors by imposing WNG ≥ 0 dB using complex weights (solid line) or real weights (dashed line); the directivity obtained using uniform weights (dotted line) is included for comparison.

First, we assess the performance of the end-fire array without oversteering when complex or real weight coefficients are used. For a given value of *d*/λ, the optimum complex weights are computed by setting ε = 0 and solving Equation (23). The optimum real weights are computed by setting ε = 0 and solving Equation (28). CVX software is employed for convex programming [20] on a common PC equipped with an Intel^®^ Core i5 CPU with 2.60 GHz of clock and 12 Gbyte of RAM, and the solution is found in less than 0.2 s. Figure 4 compares the maximum constrained directivities and illustrates the directivity obtainable with uniform weights. In this specific case, the optimum real weights only provide a directivity higher than that obtained by uniform weights for *d*/λ < 0.22, with a gain that does not exceed 2 dB. In contrast, the complex weights provide a directivity gain of approximately 5 dB over a wide interval of *d*/λ. The absolute maximum constrained directivity is obtained for *d*/λ = 0.36 and has a value of 15.3 dB. Because 10 log(*N*) is 9 dB and 10 log(*N*^2^) is 18 dB, the optimum complex weights allow for a robust directivity with an absolute maximum that is significantly higher than *N* and moderately lower than *N*^2^.

**Figure 5:**
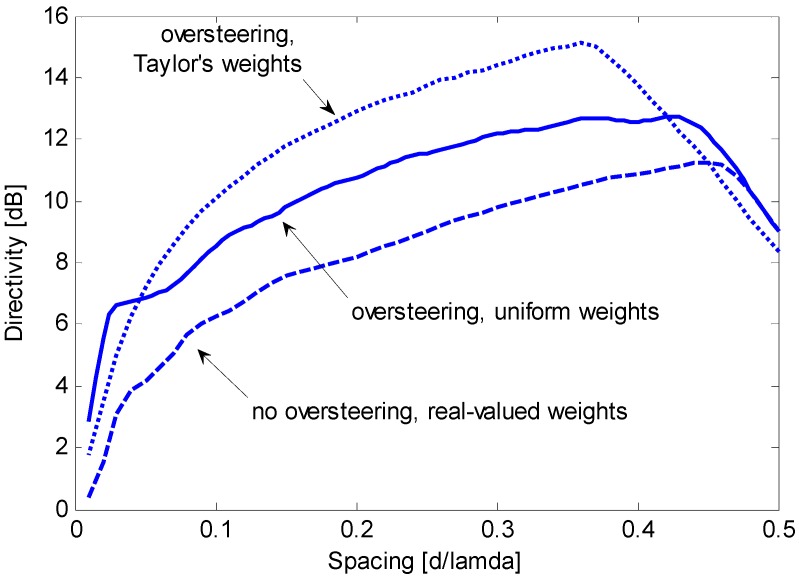
Maximum constrained directivity obtained for an end-fire array of *N* = 8 sensors by imposing WNG ≥ 0 dB using the oversteering technique with uniform weights (solid line) and Taylor’s weights (dotted line); the performance obtained without oversteering using optimum real weights is included for comparison (dashed line).

Figure 5 compares the performances obtained by the oversteering technique when the uniform and Taylor’s weighting windows are applied. The optimum value for the oversteering amount ε, where ε ϵ (0, λ/*d* − 2), is computed by the algorithm described in Section 3.3 using a predefined weight vector (uniform or Taylor’s) instead of **w***_oR_*(ε*_z_*). A discretization step for ε of 0.01 is established and a Matlab^®^ script is run on the previously mentioned PC, which results in a computation time that does not exceed 5 s.

**Figure 6:**
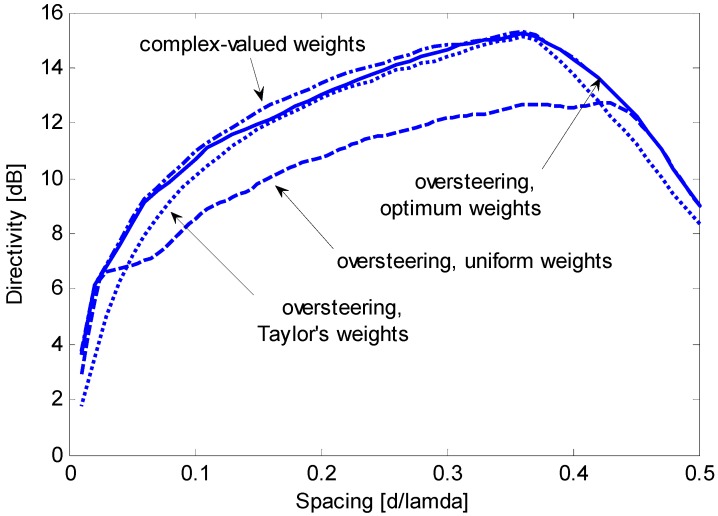
Maximum constrained directivity obtained for an end-fire array of *N* = 8 sensors by imposing WNG ≥ 0 dB using the oversteering technique with optimum weights (solid line), uniform weights (dashed line), and Taylor’s weights (dotted line); the performance without oversteering obtained with optimum complex weights is included for comparison (dash-dotted line).

**Figure 7:**
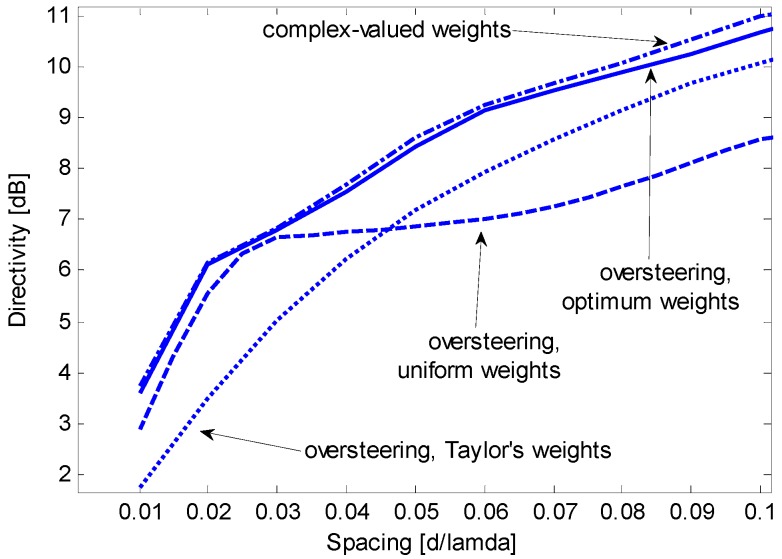
Magnification of a section from Figure 6.

**Figure 8:**
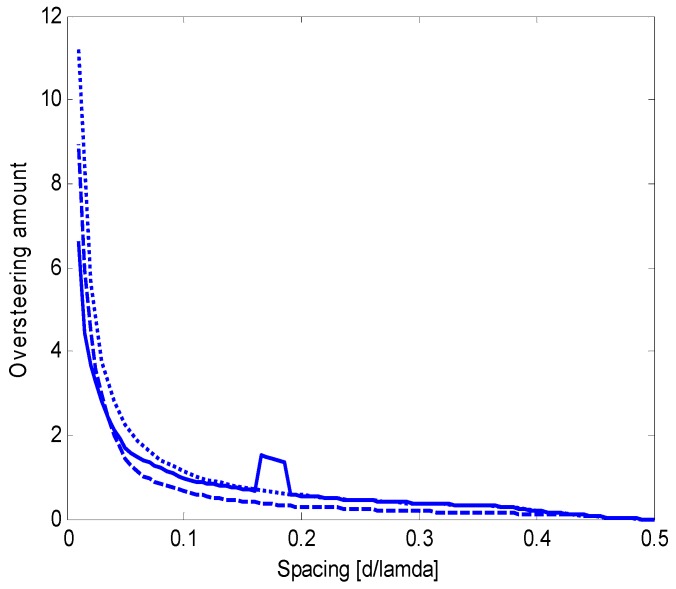
Oversteering amount, ε, that provides the maximum constrained directivity for an end-fire array of *N* = 8 transducers, which is obtained by imposing WNG ≥ 0 dB for three weighting windows: optimum weights (solid line), uniform weights (dashed line), and Taylor’s weights (dotted line).

Figure 5 shows that the oversteering technique performs better than the optimum real weights without oversteering. In particular, Taylor’s window provides advantageous directivity values over a wide interval of *d*/λ, from 0.05 to approximately 0.42. According to Figure 4 and Figure 5, the performance achieved by real weights (uniform or optimized) without oversteering is poorer than the performance achieved by complex weights or by the oversteering technique; as a result, real weights without oversteering will be disregarded in subsequent investigations. The latter is intended to assess the oversteering with the real weights that maximize the constrained directivity. For a given value of *d*/λ, the oversteering optimization and the weight computation are performed by the algorithm described in Section 3.3. A discretization step for ε of 0.01 is established, and CVX software is employed for convex programming [20] on the previously mentioned PC, which results in a computation time that does not exceed 4 min. Figure 6 presents the maximum constrained directivities obtained with the oversteering technique using three weighting windows: uniform, Taylor’s, and optimized. The absolute maximum for the constrained directivity (*i.e.*, the performance obtained with the optimum complex weights) is also included for comparison. It is possible to verify that the optimum real-valued weights always provide the best oversteering performance and that the achieved directivity is very close or equal to the absolute maximum. Although the Taylor’s weights provide results that are only slightly poorer over a significant interval of *d*/λ (from approximately 0.1 to 0.4), the optimized weights ensure the achievement of the maximum constrained directivity achievable by the oversteering technique. In this specific case, for values of *d*/λ lower than 0.1, the optimized weights provide a significant advantage over traditional weighting windows. To realize this fact, Figure 7 presents a magnification of a portion of Figure 6. Figure 8 presents the oversteering amount ε required to obtain the maximum constrained directivity using uniform, Taylor’s, and optimized weights. In general, the oversteering amount ε necessary to achieve the maximum directivity increases as the spacing *d* decreases and the allowed interval for ε increases. When *d*/λ is less than 0.04, the optimized weights achieve the maximum constrained directivity using an oversteering amount that is smaller than those with uniform and Taylor’s weights. Thus, unlike oversteering with traditional weights, the proposed optimization performs best when considering both the oversteering amount and weighting window.

**Figure 9:**
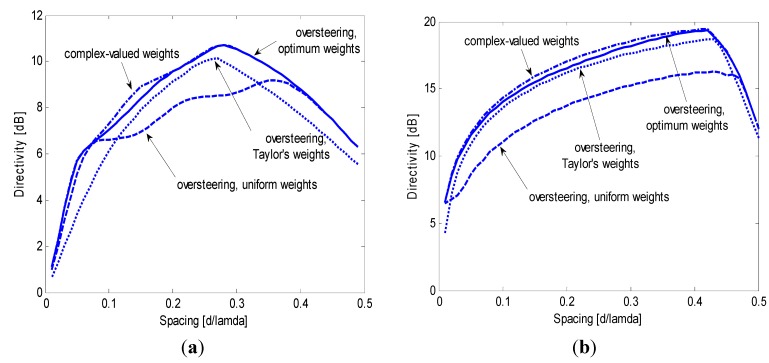
Maximum constrained directivity obtained for end-fire arrays of (**a**) *N* = 4 and (**b**) *N* = 16 sensors by imposing WNG ≥ 0 dB for the oversteering technique with optimum weights (solid line), uniform weights (dashed line), and Taylor’s weights (dotted line); the performance obtained without oversteering using optimum complex weights is included for comparison (dash-dotted line).

An assessment of the oversteering performance with respect to the number of array sensors is shown in Figure 9, where the same comparison as Figure 6 is repeated for *N* = 4 (Figure 9a) and *N* = 16 (Figure 9b). The computation times do not change considerably with respect to the aforementioned values (a moderate increase is observed also if the number of array sensor becomes greater than 16). With both four and 16 sensors, the oversteering with optimized weights yields almost the same directivity as the optimum complex weights over the entire spacing domain. Where a difference is visible, it does not exceed 0.6 dB. Oversteering with Taylor’s and uniform weights does not guarantee the same performance level: for *N* = 4, both weighting windows yield a constrained directivity that is approximately 2 dB lower than the best constrained directivity over large intervals of the spacing domain; for *N* = 16, Taylor’s window performs generally well but exhibits a 2-dB fall at the lower bound of the spacing domain, whereas the uniform weights yield a directivity that is approximately 3 dB lower than the best constrained directivity over a large portion of the spacing domain.

For *N* = 4 and *N* = 16 (as well as for *N* = 8), the oversteering with optimum weights allows for a robust directivity with an absolute maximum that is significantly higher than *N* and moderately lower than *N*^2^. The spacing value at which the maximum directivity is obtained approaches 0.5 λ as *N* increases.

**Figure 10:**
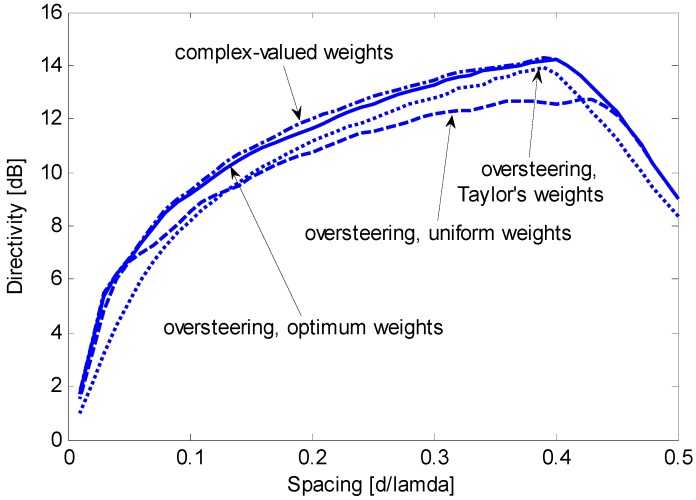
Maximum constrained directivity obtained for an end-fire array of *N* = 8 sensors by imposing WNG ≥ 5 dB for the oversteering technique with optimum weights (solid line), uniform weights (dashed line), and Taylor’s weights (dotted line); the performance obtained without oversteering using optimum complex weights is included for comparison (dash-dotted line).

A final assessment considers robustness against mismatches of the sensor characteristics. A more severe bound can be imposed if the robustness provided by a WNG ≥ 0 dB is not sufficient. Figure 10 presents the same comparison as Figure 6 for the case when the WNG is imposed to be greater than 5 dB. In this case, the maximum achievable directivity is generally 1–2 dB lower than that obtained by imposing WNG ≥ 0 dB. Although the performance graphs are now closer to each other, the oversteering with optimum weights is still the only technique that yields a constrained directivity very similar to that of the optimum complex weights over the entire spacing domain. The absolute maximum of the constrained directivity is now obtained for *d*/λ = 0.39 and has a value of 14.3 dB (compared to 15.3 dB obtained imposing WNG ≥ 0 dB). Because a WNG of 5 dB ensures a very robust design (for an eight-element array, the absolute maximum of the WNG is 9 dB), we conclude that, in this extreme case, the oversteering with optimum weights also provides a robust directivity with an absolute maximum between *N* and *N*^2^.

### 4.2. System Implementation

An analysis of Equation (7) reveals that for any ES linear array, the signal delays used with the oversteering technique differ from each other by an integer multiple of the quantity Δ, defined as follows:

(30) $$\text{Δ}=\frac{d(1+\text{ε})}{c}$$

Thus, if the sampling frequency *f_s_* is set to 1/Δ or to an integer multiple of 1/Δ, all of the delays to be applied to the received signals can be implemented in an easy and exact manner by shifting the signal of a given number of samples. Thus, before the beamforming sum, each signal should be multiplied by a real gain factor and shifted by an integer number of samples.

To verify the viability of this procedure, we can consider a linear array of microphones spaced 3.4 cm each other, working at a nominal frequency of 3.4 kHz (*i.e.*, *d* = 0.34 λ). In this case, the microphone signals should be sampled at a frequency equal to or lower than 10 kHz, depending on the value of ε.

**Figure 11:**
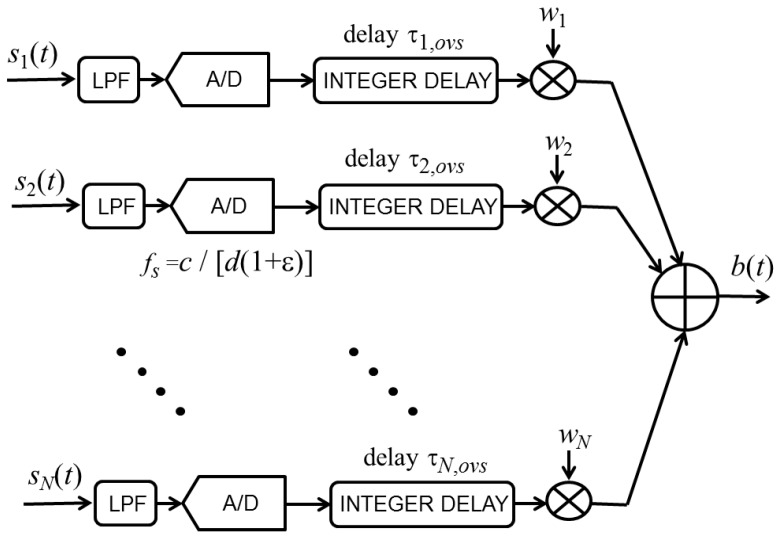
Schematic of oversteered end-fire beamformer for a suitable sampling frequency *f_s_*.

Figure 11 displays a schematic of the implementation of this beamforming technique. After low-pass filtering (LPF) to avoid aliasing, the *N* input signals are digitized through analog-to-digital (A/D) converters that sample at a frequency *f_s_* = 1/Δ = *c*/[*d*(1 + ε)]. If *f_s_* is lower than the Nyquist rate, an integer multiple of *f_s_* can be set as the sampling frequency. The *n*th delay τ*_n,ovs_* given in Equation (7) can be obtained very efficiently by shifting the *n*th signal by a given number of samples using a digital integer delay line. Before performing the final sum that generates the output signal *b*(*t*), the *n*th signal is multiplied by the real weight coefficient *w_n_*. If optimum oversteering performance is desired, the weights *w_n_* should be computed by solving Equation (28). Otherwise, the weights can be derived from a traditional weighting window, such as Taylor’s window.

The same simple implementation cannot be applied for the complex weights because the term φ*_n_* in Equation (6) depends on the index *n*. The implementation of end-fire beamforming with complex weights requires the precise implementation of delays that are not integer multiples of the sampling period or the transformation of the received signals in their analytic versions and the processing of complex signals. Both these options are more involved than the implementation of the oversteered end-fire array, needing a processing architecture that requires additional money, resources, and space.

**Figure 12:**
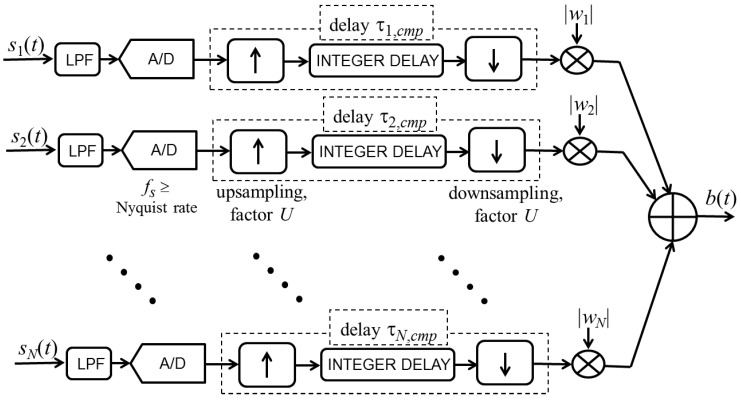
Schematic of end-fire beamformer using complex weights, where delays are implemented using digital fractional delay lines.

Of the two options for conducting end-fire beamforming with complex weights, the use of real signals is most similar to oversteering. This beamforming technique is mathematically defined in Equation (3), and a potential implementation scheme is shown in Figure 12. After low-pass filtering (LPF) to avoid aliasing, the *N* input signals are digitized through analog-to-digital (A/D) converters that sample at a frequency *f_s_*, which is greater than or equal to the Nyquist rate. Unlike the oversteering case, the deployment of the delays τ*_n,cmp_* given in Equation (6) requires digital fractional delay lines. For each signal, the commonly used implementation scheme includes an interpolator that upsamples the signal by a suitable factor *U*, an integer delay line that approximates τ*_n,cmp_* by shifting the signal by a given number of samples at the higher rate, and a decimator that downsamples the signal by the factor *U*. Before performing the final sum that generates the output signal *b*(*t*), the *n*th signal should be multiplied by the modulus of the complex weight coefficient *w_n_*. To achieve the maximum constrained directivity, the weights *w_n_* should be computed by solving Equation (23). An alternative to using the interpolator and decimator is to sample and process the input signals directly at the higher rate (*i.e.*, *f_s_U*). However, the example described in the next subsection demonstrates that such a sampling rate is much higher than that required for the oversteering case.

The comparison of Figure 11 and Figure 12 and consideration of the previously mentioned observations confirm that the implementation of the end-fire beamformer with complex weights is more complicated than the implementation of the oversteered beamformer.

### 4.3. Simulated Testing of an Array Design

To test the performance of the beamformers considered in this paper, we consider a linear array of eight microphones spaced 2.5 cm apart. We consider that a plane wave with a frequency of 680 Hz impinges on the array at an arrival angle that varies from −90° to 90°. Random mismatches among the microphone responses are introduced according to specified statistics. For each arrival angle, the electrical signals produced by the array sensors are digitized and processed according to the schematics shown in Figure 11 and Figure 12. The energy of the obtained beam signal is used to compose the actual beam power pattern |*B_a_*(*u*)|^2^ of the simulated array.

**Figure 13:**
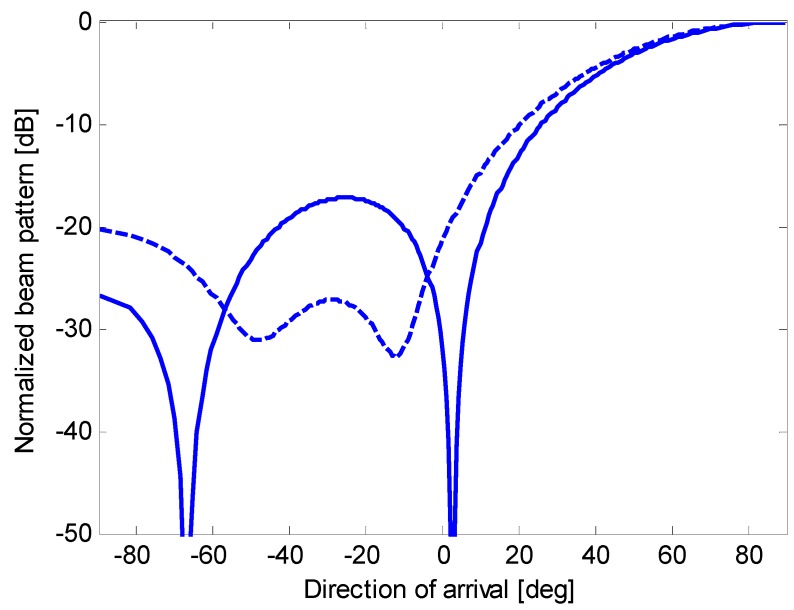
Nominal beam pattern (solid line) and actual beam pattern (dashed line) for beamforming with optimum complex weights applied to an end-fire array of eight microphones.

**Figure 14:**
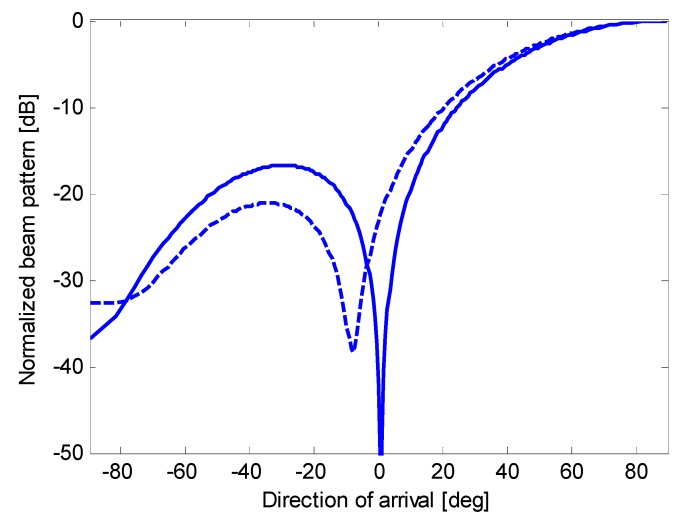
Nominal beam pattern (solid line) and actual beam pattern (dashed line) for oversteered beamforming with optimum real weights applied to an end-fire array of eight microphones.

**Figure 15:**
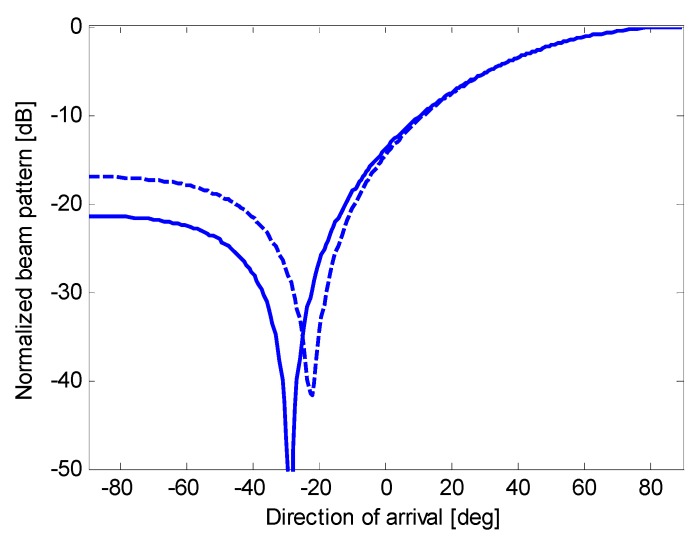
Nominal beam pattern (solid line) and actual beam pattern (dashed line) for oversteered beamforming with Taylor’s weights applied to an end-fire array of eight microphones.

At a frequency of 680 Hz, the inter-element spacing *d* is equal to 0.05 λ; thus, the nominal array performance for a WNG greater than or equal to 0 dB is as follows. Figure 7 shows that the maximum constrained directivity is 8.62 dB using complex weights and 8.43 dB for oversteering using optimized weights. In the latter case, Figure 8 shows that the oversteering amount should be ε = 1.71. For comparison, if oversteering is used with Taylor’s weights, the maximum constrained directivity is 7.20 dB and is achieved for an oversteering amount ε = 2.25. The nominal beam patterns for these three options are shown in Figure 13, Figure 14 and Figure 15.

The performance is assessed using two subsequent tests: in the first test, the sampling frequency (and the upsampling factor) needed to achieve an actual beam pattern close to the nominal beam pattern is determined. In the second test (see the following subsection), a statistical investigation is used to assess the robustness of the beamforming performance against mismatches in the sensor responses.

In the first test, to clearly determine the relation between the sampling frequency and the beam pattern accuracy requires the assumption of ideal microphones (*i.e.*, sensors with perfectly matched responses) and the use of a metric for the beam pattern distortion. The metric is developed by introducing the percentage error ∑, which is defined as the normalized distance between the actual beam power pattern |*B_a_*(*u*)|^2^ and the nominal beam power pattern |*B*(*u*)|^2^:

(31) $$\Sigma =100\;\frac{\int_{-1}^{1}\left|\;{\left|B\left(u\right)\right|}^{2}-{\left|B_{a}\left(u\right)\right|}^{2}\right|\;du}{\int_{-1}^{1}{\left|B\left(u\right)\right|}^{2}du}$$

Obviously, both of the beam power patterns that are substituted into this equation must to be normalized functions.

The oversteering amount that optimizes the performance should be used to set the sampling frequency *f_s_* = 5.018 kHz for the oversteered beamforming with optimized weights. Using this specific value of *f_s_* for the processing outlined in Figure 11 results in an actual beam pattern that is identical to the nominal beam pattern. However, it is not necessary to acquire exactly 5018 samples per second to obtain good results: for instance, if the sampling frequency is set to 5 kHz or 5.05 kHz, the percentage error, ∑, does not exceed 1.9%. Similar conclusions are obtained for oversteered beamforming using Taylor’s weights. A zero error is obtained for a sampling frequency of 4.185 kHz; however, at frequencies around this value, the beam pattern is not significantly altered.

**Figure 16:**
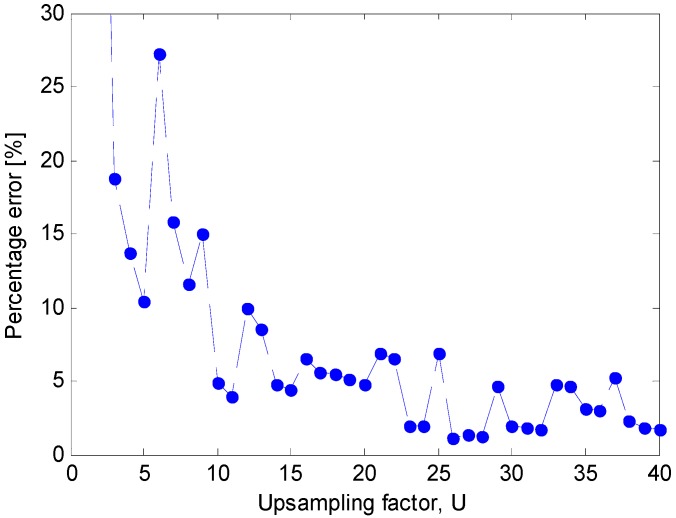
Distortion of beam pattern (measured in terms of the percentage error ∑) for beamforming with optimum complex weights *versus* the upsampling factor *U*.

**Figure 17:**
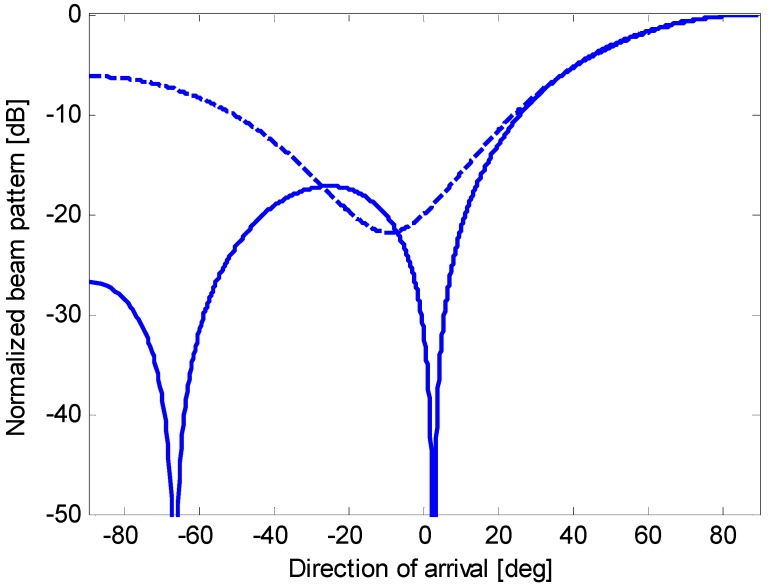
Nominal beam pattern (solid line) and actual beam pattern (dashed line) for beamforming with optimum complex weights when *f_s_* = 5 kHz and *U* = 1 for an end-fire array of 8 microphones.

For beamforming using optimum complex weights, a sampling frequency *f_s_* = 1.5 kHz is set that is slightly higher than the Nyquist rate. Figure 16 shows the measured percentage error, ∑, when the upsampling factor *U* is increased up to 40. To obtain an error, ∑, that is lower than 5%, an upsampling factor *U* = 10 is necessary. Instead, if an error, ∑, lower than 2% is desired, the minimum upsampling factor is *U* = 23. If a sampling frequency similar to the sampling frequency of the oversteered case is adopted without upsampling (*i.e.*, *f_s_* = 5 kHz and *U* = 1), an error ∑equal to 22.7% is achieved and the actual beam pattern exhibits major distortions, as shown in Figure 17.

The information on the minimum upsampling factor that is needed to assure a small error, joined with the implementation scheme in Figure 12, provides a clear indication about the additional complexity that the beamforming with optimum complex weights requires.

### 4.4. Statistical Assessment

To start the statistical investigation with a sufficiently accurate beam pattern, the sampling frequency is set as follows: 1.5 kHz, with an upsampling factor *U* = 10 for the optimum complex weights; 5 kHz for oversteering with optimized weights; and 4.2 kHz for oversteering with Taylor’s weights.

Mismatches in the sensor characteristics are typically modeled [2,21] by multiplying the response of the microphones by a random complex variable. The random variable *A_n_*, where *A_n_* = *a_n_* exp(–*j*γ*_n_*), is introduced to model the gain *a_n_* and the phase γ*_n_* of the response of the *n*th microphone. We assume that all of the random variables *A_n_*, where *n* = 1, …, *N*, can be described by the same probability density function (PDF) *f_A_*(*A*). Moreover, *a_n_* and γ*_n_* are assumed to be independent random variables such that the joint PDF is separable, *f_A_*(*A*) = *f_a_*(*a*) *f*γ(γ), where *f_a_*(*a*) is the PDF of the gain, and *f*γ(γ) is the PDF of the phase. To investigate the robustness of the array performance, the PDFs of the microphone gain *a_n_* and the phase γ*_n_* are assumed to be Gaussian variables with mean values of 1 and 0, respectively, and with standard deviations of 0.1 and 0.07 rad, respectively. The standard deviation values are significantly higher than the experimentally measured values for commercial microphone arrays [22,23,24]. The reason for testing the array designs considered in this paper under such critical conditions is to evaluate the performance that can be achieved by deploying low-cost microphones.

Figure 13, Figure 14 and Figure 15 show the actual beam patterns obtained if we consider only one realization of the random variables *A_n_*, *n* = 1, …, *N*, (which occurs in a given physical implementation of the array). These beam patterns are computed by simulating the reception and processing of a plane wave following the procedure described at the beginning of the previous subsection. The related directivities are as follows: 8.12 dB for the optimum complex weights (against a nominal *D* of 8.62 dB); 8.09 dB for oversteering with optimized weights (against a nominal *D* of 8.43 dB); and 7.19 dB for oversteering with Taylor’s weights (against a nominal *D* of 7.20 dB). Thus, the reduction in the directivity is a minimum for oversteering with Taylor’s weights and a maximum using the optimum complex weights. However, these results only serve as an example.

To ensure that the performance analysis is statistically relevant, the actual beam patterns are evaluated for 10^4^ realizations of the random variables *A_n_*, *n* = 1, …, *N*. The directivities of these beam patterns are computed and used to derive the sample mean and the standard deviation and to trace the worst-case value. The results are reported in Table 1. Note that the mean directivity is approximately 0.3 dB lower than the nominal directivity for both oversteering with optimized weights and using optimum complex weights. This difference is reduced to 0.2 dB for oversteering with Taylor’s weights. The standard deviations for oversteering with optimized weights and Taylor’s weights are approximately equal and lower than that obtained using optimum complex weights. The worst-case value of the actual directivity is approximately 2.3 dB lower than the nominal directivity for both oversteering with optimized weights and using optimum complex weights. This difference is reduced to 2.0 dB for oversteering with Taylor’s weights.

**Table 1:** Nominal directivity compared with the sample mean and worst-case value of the actual directivity for an end-fire array of 8 microphones and 10^4^ realizations of the mismatches; Optimum complex weights, oversteering with optimum weights, and oversteering with Taylor’s weights are considered; The standard deviation (as a linear scale) of the actual directivity is included.

	Optimum Complex Weights	Oversteering, Optimum Weights	Oversteering, Taylor’s Weights
Nominal directivity	8.62 dB	8.43 dB	7.20 dB
Mean directivity	8.32 dB	8.12 dB	7.01 dB
Stand. Deviation	0.64	0.49	0.46
Worst-case directivity	6.27 dB	6.12 dB	5.20 dB

Despite the large magnitude assumed for the microphone mismatches, the three considered end-fire beamformers exhibit satisfactory robustness because of the constraint imposed on the WNG value. Oversteering with optimized weights and using optimum complex weights yield similar mean values and worst-case values for the actual directivity. However, the oversteered beamformer has a lower variance. The robustness of oversteering with Taylor’s weights is slightly better than the other cases most likely because the related directivity is the lowest for oversteering with Taylor’s weights. Overall, the advantages offered by implementing the oversteered beamformer (with optimized or Taylor’s weights) are not compromised by the performance reduction from the sensor mismatches because a similar reduction is also observed for the beamformer using optimum complex weights.

Although this section does not explicitly consider positioning errors, it is well known that they can be considered as phase errors whose standard deviation depends on the wavelength. In particular, if the transducer positions are affected by independent, zero-mean, Gaussian errors, with equal variances along the three Cartesian axes, it is possible to model the position error as a phase error whose standard deviation is equal to the standard deviation of the position error multiplied by the wavenumber [2,25]. Overall, the impact of a given position error increases as the wavelength decreases.

## 5. Conclusions

In this paper, we proposed a method to compute the amount of oversteering and the related vector of real weight coefficients that provide the maximum constrained directivity that can be obtained through the oversteering technique applied to a linear end-fire array of ES sensors. The constraint is related to the WNG and ensures the robustness of the beam pattern against mismatches of the sensor characteristics. The numerical simulation results confirmed that the optimized performance is robust even in the presence of considerable sensor mismatches. Moreover, we verified that for every spacing value, the constrained directivity achieved by the proposed method is higher than the directivity obtained through oversteering with traditional weighting windows and very close to the absolute maximum for the constrained directivity. The latter characteristic requires complex weight coefficients that make the processing system more involved. In contrast, a linear oversteered end-fire array of ES sensors can be easily implemented by selecting an adequate sampling frequency, scaling each received signal by a real gain value, shifting each signal of an integer number of samples, and summing all of the signals together.

More generally, we have investigated the maximum constrained directivity of an end-fire array as a function of the inter-element spacing for different numbers of sensors and WNG lower bounds. We reported a constrained directivity between *N* and *N*^2^ that was obtained at a spacing value *d* < 0.5 λ by using complex weights or oversteering with real weights. In the examined cases, these two techniques provided a directivity equal to *N* when the spacing does not exceed 0.1 λ and when WNG ≥ 0 dB.

Future activities will be focused to: assess the potential of the oversteering technique in approaching the maximum generalized directivity (*i.e.*, the directivity of the mean beam power pattern, computed using the statistics of the array errors, as recently proposed in [25]); investigate the opportunity of introducing the oversteering concept in the context of the differential microphone arrays, according to the latest advancements described in [26].
